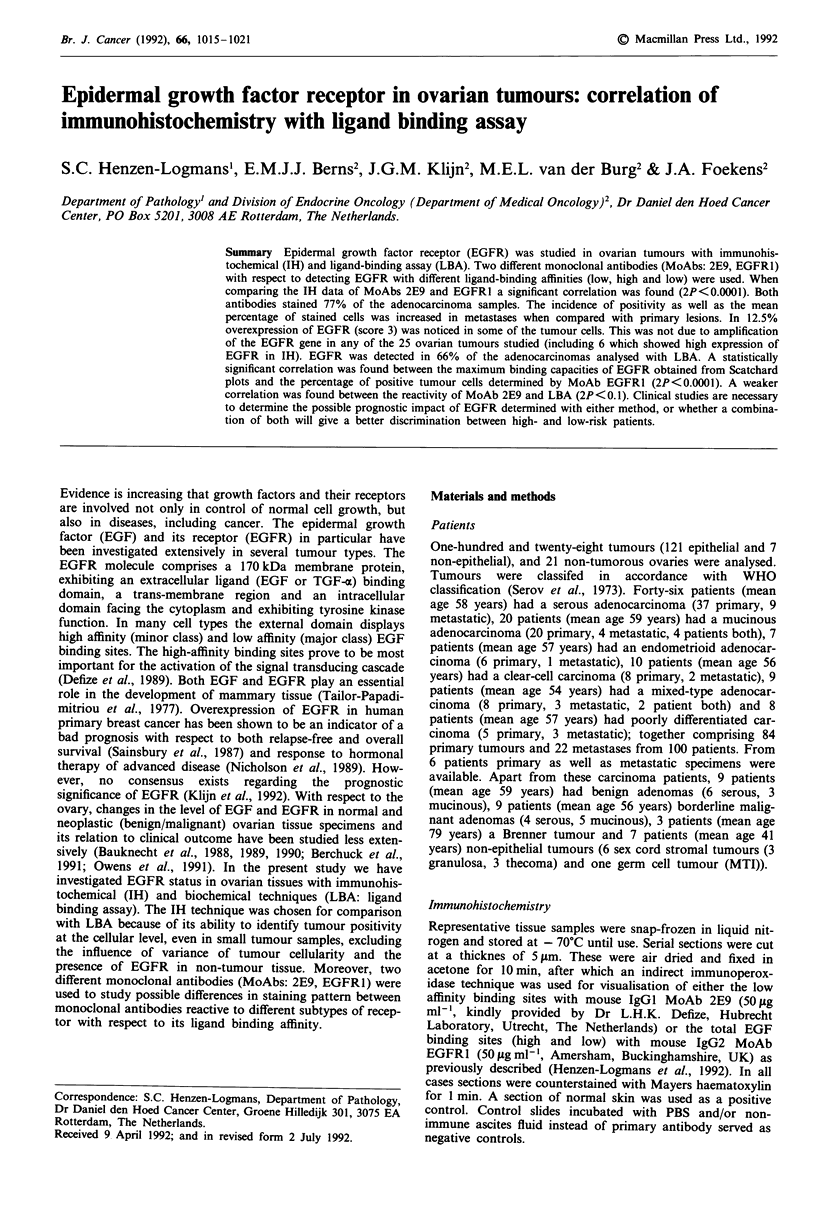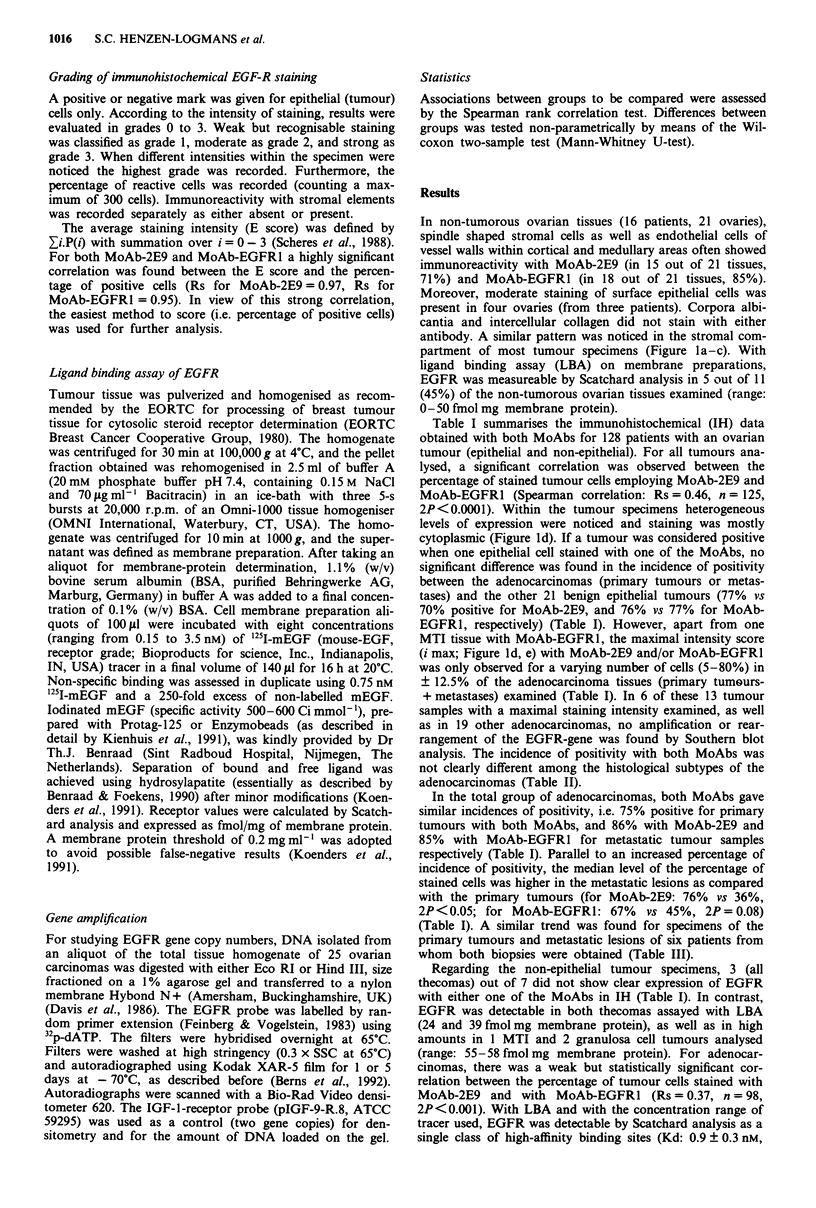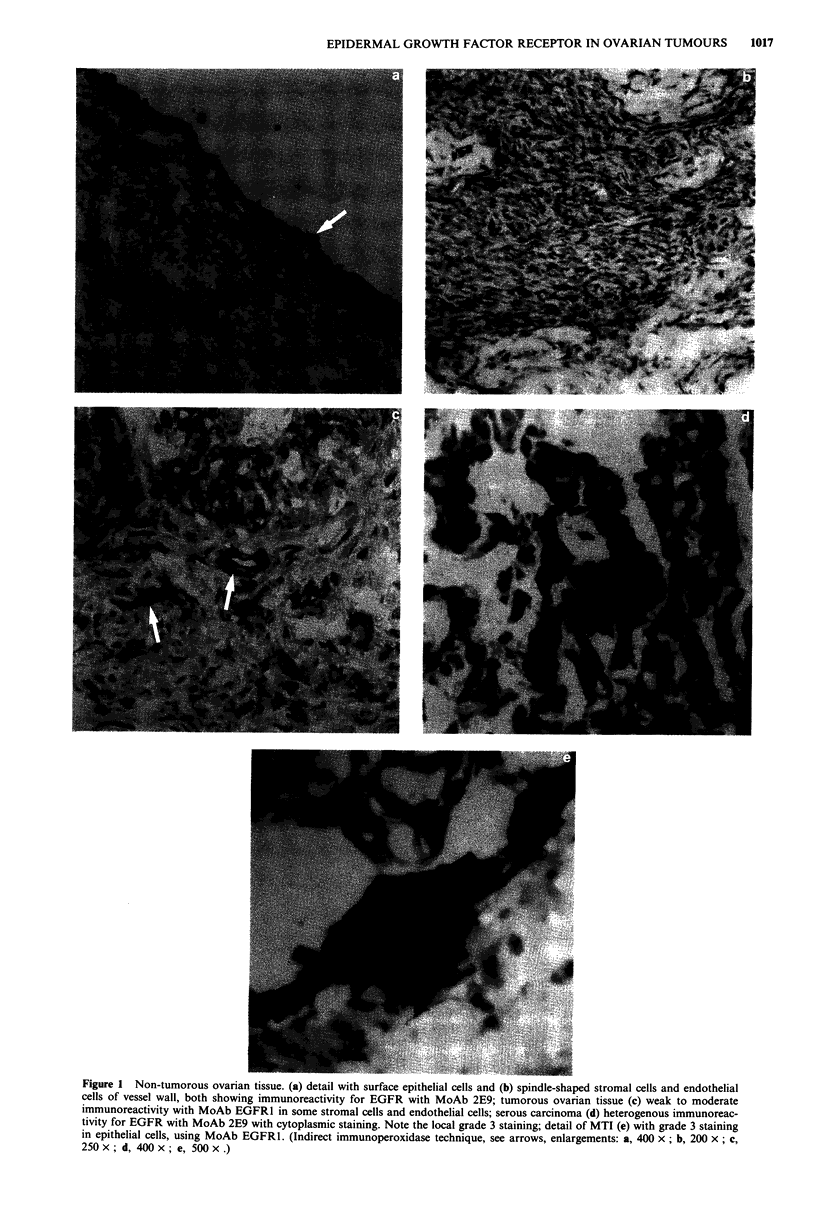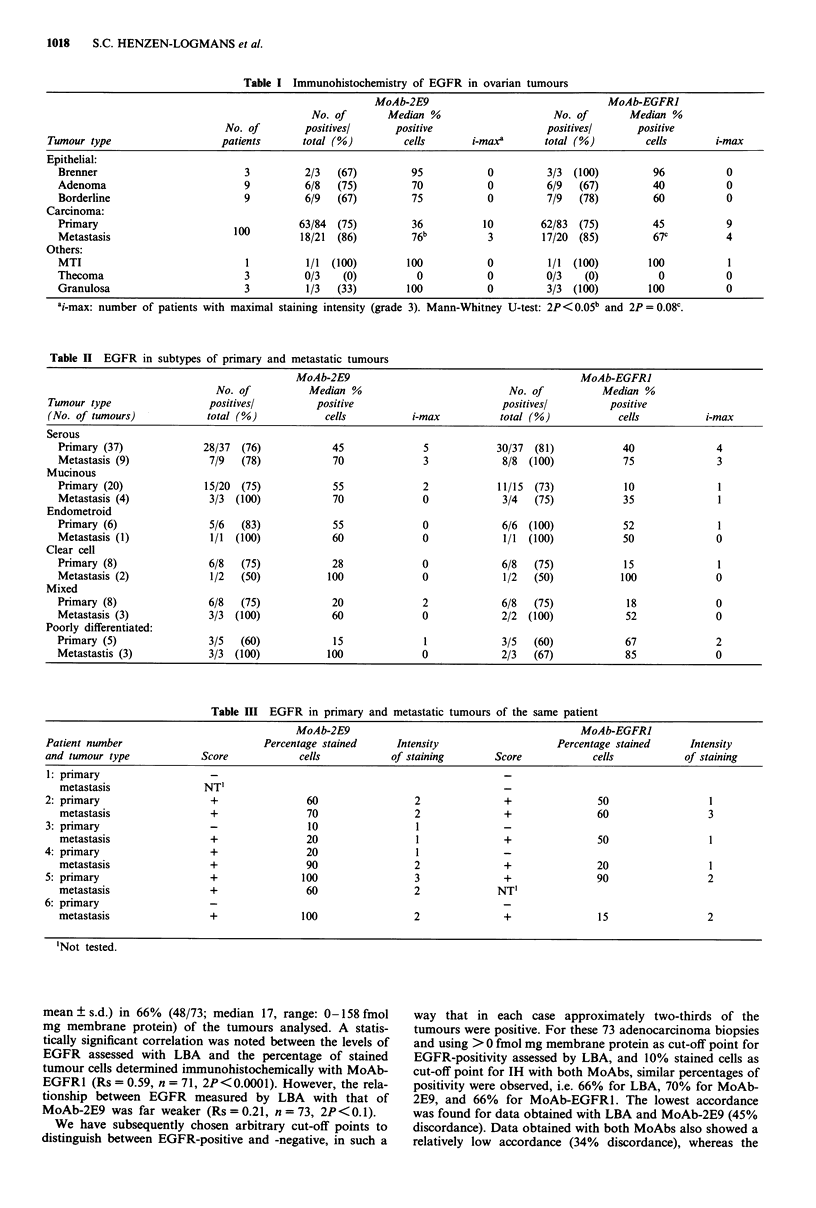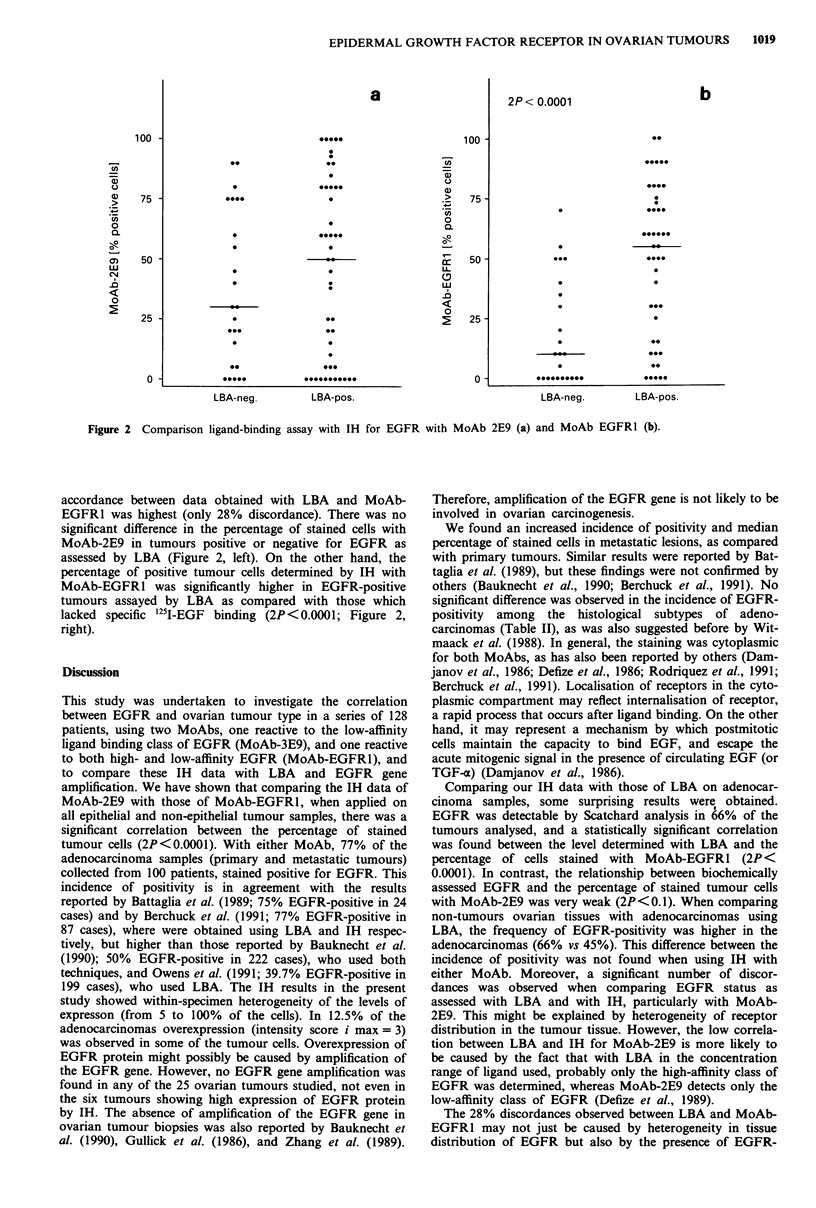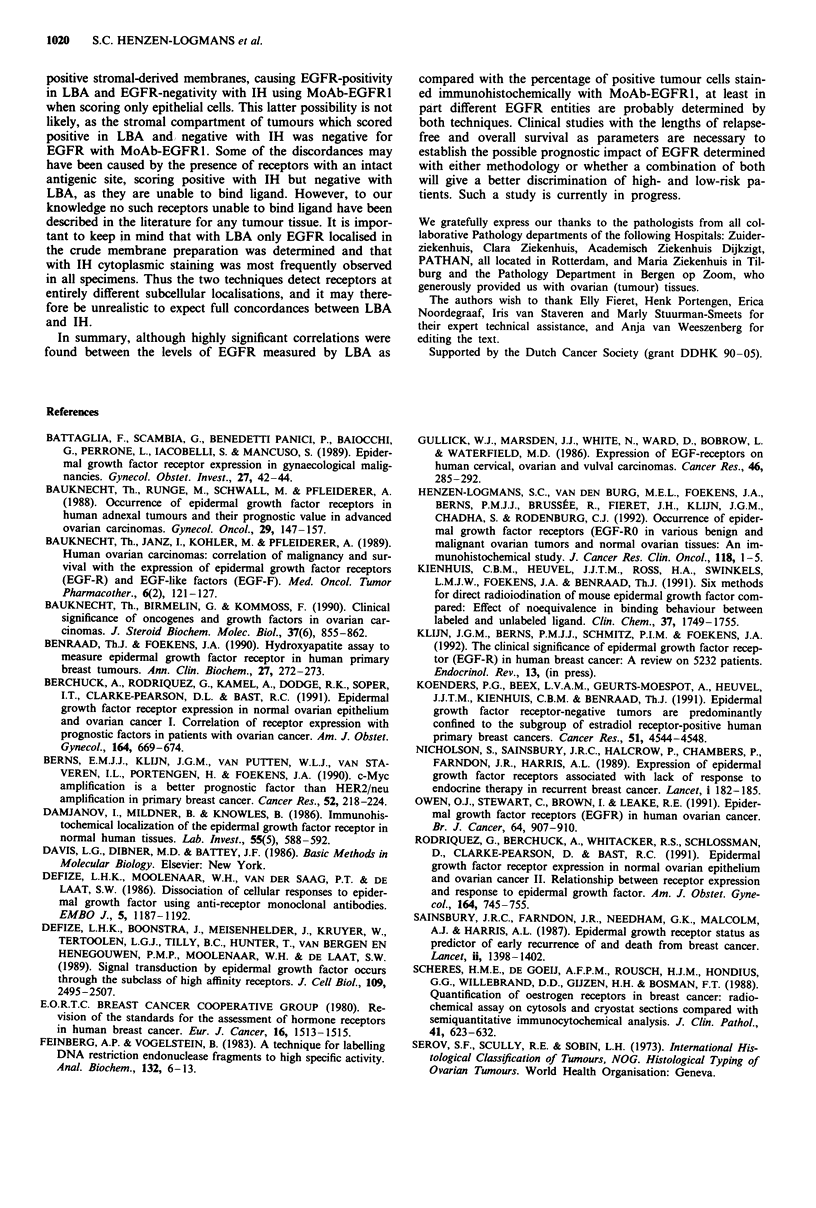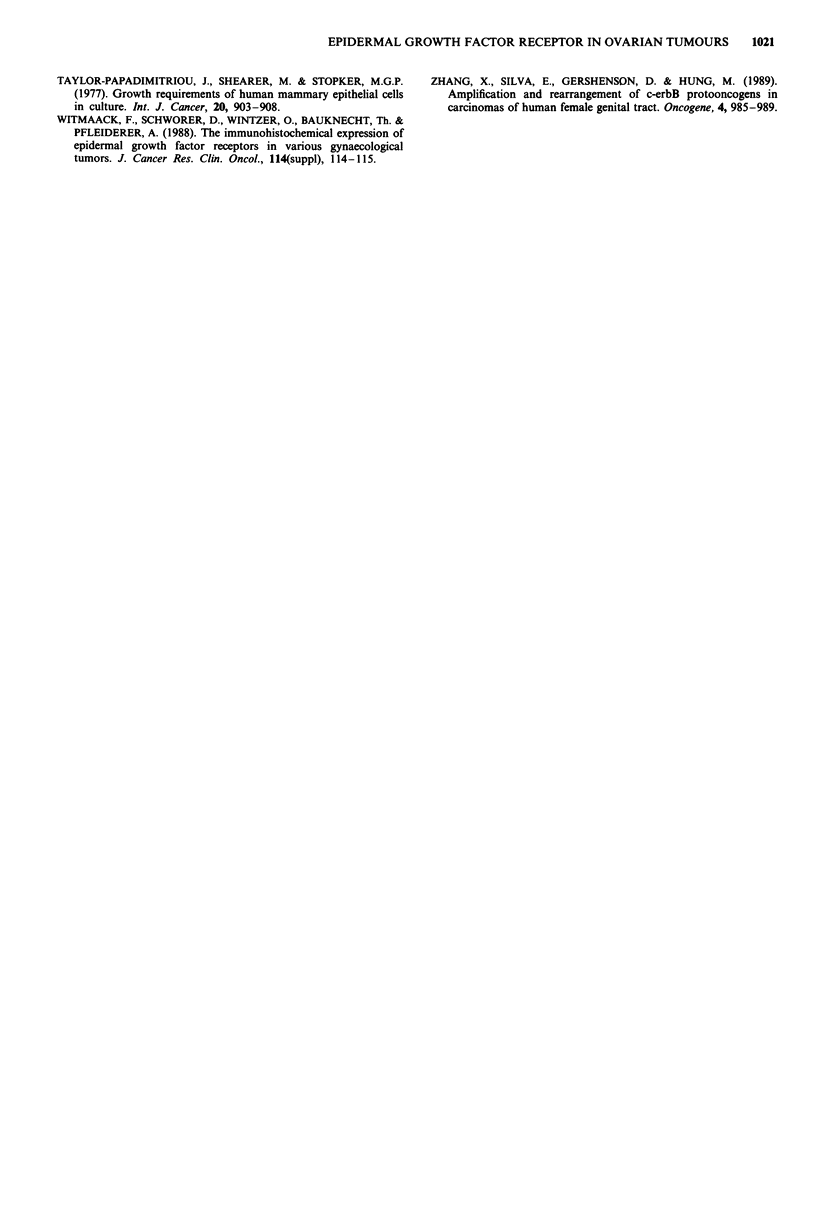# Epidermal growth factor receptor in ovarian tumours: correlation of immunohistochemistry with ligand binding assay.

**DOI:** 10.1038/bjc.1992.403

**Published:** 1992-12

**Authors:** S. C. Henzen-Logmans, E. M. Berns, J. G. Klijn, M. E. van der Burg, J. A. Foekens

**Affiliations:** Department of Pathology, Dr Daniel den Hoed Cancer Center, Rotterdam, The Netherlands.

## Abstract

**Images:**


*Br. J. Cancer* (1992), **66**, 1015–1021 © Macmillan Press Ltd., 1992

### Epidermal growth factor receptor in ovarian tumours: correlation of immunohistochemistry with ligand binding assay

S.C. Henzen-Logmans[1], E.M.J.J. Berns[2], J.G.M. Klijn[2], M.E.L. van der Burg[2] & J.A. Foekens[2]

*Department of Pathology[1] and Division of Endocrine Oncology (Department of Medical Oncology)[2], Dr Daniel den Hoed Cancer Center, PO Box 5201, 3008 AE Rotterdam, The Netherlands.*

**Summary** Epidermal growth factor receptor (EGFR) was studied in ovarian tumours with immunohistochemical (IH) and ligand-binding assay (LBA). Two different monoclonal antibodies (MoAbs: 2E9, EGFR1) with respect to detecting EGFR with different ligand-binding affinities (low, high and low) were used. When comparing the IH data of MoAbs 2E9 and EGFR1 a significant correlation was found ($2P<0.0001$). Both antibodies stained 77% of the adenocarcinoma samples. The incidence of positivity as well as the mean percentage of stained cells was increased in metastases when compared with primary lesions. In 12.5% overexpression of EGFR (score 3) was noticed in some of the tumour cells. This was not due to amplification of the EGFR gene in any of the 25 ovarian tumours studied (including 6 which showed high expression of EGFR in IH). EGFR was detected in 66% of the adenocarcinomas analysed with LBA. A statistically significant correlation was found between the maximum binding capacities of EGFR obtained from Scatchard plots and the percentage of positive tumour cells determined by MoAb EGFR1 ($2P<0.0001$). A weaker correlation was found between the reactivity of MoAb 2E9 and LBA ($2P<0.1$). Clinical studies are necessary to determine the possible prognostic impact of EGFR determined with either method, or whether a combination of both will give a better discrimination between high- and low-risk patients.

Evidence is increasing that growth factors and their receptors are involved not only in control of normal cell growth, but also in diseases, including cancer. The epidermal growth factor (EGF) and its receptor (EGFR) in particular have been investigated extensively in several tumour types. The EGFR molecule comprises a 170 kDa membrane protein, exhibiting an extracellular ligand (EGF or TGF-α) binding domain, a trans-membrane region and an intracellular domain facing the cytoplasm and exhibiting tyrosine kinase function. In many cell types the external domain displays high affinity (minor class) and low affinity (major class) EGF binding sites. The high-affinity binding sites prove to be most important for the activation of the signal transducing cascade (Defize *et al.*, 1989). Both EGF and EGFR play an essential role in the development of mammary tissue (Tailor-Papadimitriou *et al.*, 1977). Overexpression of EGFR in human primary breast cancer has been shown to be an indicator of a bad prognosis with respect to both relapse-free and overall survival (Sainsbury *et al.*, 1987) and response to hormonal therapy of advanced disease (Nicholson *et al.*, 1989). However, no consensus exists regarding the prognostic significance of EGFR (Klijn *et al.*, 1992). With respect to the ovary, changes in the level of EGF and EGFR in normal and neoplastic (benign/malignant) ovarian tissue specimens and its relation to clinical outcome have been studied less extensively (Bauknecht *et al.*, 1988, 1989, 1990; Berchuck *et al.*, 1991; Owens *et al.*, 1991). In the present study we have investigated EGFR status in ovarian tissues with immunohistochemical (IH) and biochemical techniques (LBA: ligand binding assay). The IH technique was chosen for comparison with LBA because of its ability to identify tumour positivity at the cellular level, even in small tumour samples, excluding the influence of variance of tumour cellularity and the presence of EGFR in non-tumour tissue. Moreover, two different monoclonal antibodies (MoAbs: 2E9, EGFR1) were used to study possible differences in staining pattern between monoclonal antibodies reactive to different subtypes of receptor with respect to its ligand binding affinity.

Correspondence: S.C. Henzen-Logmans, Department of Pathology, Dr Daniel den Hoed Cancer Center, Groene Hilledijk 301, 3075 EA Rotterdam, The Netherlands.
Received 9 April 1992; and in revised form 2 July 1992.

### Materials and methods

### *Patients*

One-hundred and twenty-eight tumours (121 epithelial and 7 non-epithelial), and 21 non-tumorous ovaries were analysed. Tumours were classifed in accordance with WHO classification (Serov *et al.*, 1973). Forty-six patients (mean age 58 years) had a serous adenocarcinoma (37 primary, 9 metastatic), 20 patients (mean age 59 years) had a mucinous adenocarcinoma (20 primary, 4 metastatic, 4 patients both), 7 patients (mean age 57 years) had an endometrioid adenocarcinoma (6 primary, 1 metastatic), 10 patients (mean age 56 years) had a clear-cell carcinoma (8 primary, 2 metastatic), 9 patients (mean age 54 years) had a mixed-type adenocarcinoma (8 primary, 3 metastatic, 2 patient both) and 8 patients (mean age 57 years) had poorly differentiated carcinoma (5 primary, 3 metastatic); together comprising 84 primary tumours and 22 metastases from 100 patients. From 6 patients primary as well as metastatic specimens were available. Apart from these carcinoma patients, 9 patients (mean age 59 years) had benign adenomas (6 serous, 3 mucinous), 9 patients (mean age 56 years) borderline malignant adenomas (4 serous, 5 mucinous), 3 patients (mean age 79 years) a Brenner tumour and 7 patients (mean age 41 years) non-epithelial tumours (6 sex cord stromal tumours (3 granulosa, 3 thecoma) and one germ cell tumour (MTI)).

### *Immunohistochemistry*

Representative tissue samples were snap-frozen in liquid nitrogen and stored at − 70°C until use. Serial sections were cut at a thicknes of 5 μm. These were air dried and fixed in acetone for 10 min, after which an indirect immunoperoxidase technique was used for visualisation of either the low affinity binding sites with mouse IgG1 MoAb 2E9 (50 μg $ml^{-1}$, kindly provided by Dr L.H.K. Defize, Hubrecht Laboratory, Utrecht, The Netherlands) or the total EGF binding sites (high and low) with mouse IgG2 MoAb EGFR1 (50 μg $ml^{-1}$, Amersham, Buckinghamshire, UK) as previously described (Henzen-Logmans *et al.*, 1992). In all cases sections were counterstained with Mayers haematoxylin for 1 min. A section of normal skin was used as a positive control. Control slides incubated with PBS and/or non-immune ascites fluid instead of primary antibody served as negative controls.

*Grading of immunohistochemical EGF-R staining*

A positive or negative mark was given for epithelial (tumour) cells only. According to the intensity of staining, results were evaluated in grades 0 to 3. Weak but recognisable staining was classified as grade 1, moderate as grade 2, and strong as grade 3. When different intensities within the specimen were noticed the highest grade was recorded. Furthermore, the percentage of reactive cells was recorded (counting a maximum of 300 cells). Immunoreactivity with stromal elements was recorded separately as either absent or present.

The average staining intensity (E score) was defined by $\sum i.P(i)$ with summation over $i = 0 - 3$ (Scheres *et al.*, 1988). For both MoAb-2E9 and MoAb-EGFR1 a highly significant correlation was found between the E score and the percentage of positive cells (Rs for MoAb-2E9 = 0.97, Rs for MoAb-EGFR1 = 0.95). In view of this strong correlation, the easiest method to score (i.e. percentage of positive cells) was used for further analysis.

*Ligand binding assay of EGFR*

Tumour tissue was pulverized and homogenised as recommended by the EORTC for processing of breast tumour tissue for cytosolic steroid receptor determination (EORTC Breast Cancer Cooperative Group, 1980). The homogenate was centrifuged for 30 min at 100,000 *g* at 4°C, and the pellet fraction obtained was rehomogenised in 2.5 ml of buffer A (20 mM phosphate buffer pH 7.4, containing 0.15 M NaCl and 70 $\mu g\ ml^{-1}$ Bacitracin) in an ice-bath with three 5-s bursts at 20,000 r.p.m. of an Omni-1000 tissue homogeniser (OMNI International, Waterbury, CT, USA). The homogenate was centrifuged for 10 min at 1000 *g*, and the supernatant was defined as membrane preparation. After taking an aliquot for membrane-protein determination, 1.1% (w/v) bovine serum albumin (BSA, purified Behringwerke AG, Marburg, Germany) in buffer A was added to a final concentration of 0.1% (w/v) BSA. Cell membrane preparation aliquots of 100 $\mu l$ were incubated with eight concentrations (ranging from 0.15 to 3.5 nM) of $^{125}$I-mEGF (mouse-EGF, receptor grade; Bioproducts for science, Inc., Indianapolis, IN, USA) tracer in a final volume of 140 $\mu l$ for 16 h at 20°C. Non-specific binding was assessed in duplicate using 0.75 nM $^{125}$I-mEGF and a 250-fold excess of non-labelled mEGF. Iodinated mEGF (specific activity 500–600 Ci $mmol^{-1}$), prepared with Protag-125 or Enzymobeads (as described in detail by Kienhuis *et al.*, 1991), was kindly provided by Dr Th.J. Benraad (Sint Radboud Hospital, Nijmegen, The Netherlands). Separation of bound and free ligand was achieved using hydrosylapatite (essentially as described by Benraad & Foekens, 1990) after minor modifications (Koenders *et al.*, 1991). Receptor values were calculated by Scatchard analysis and expressed as fmol/mg of membrane protein. A membrane protein threshold of 0.2 mg $ml^{-1}$ was adopted to avoid possible false-negative results (Koenders *et al.*, 1991).

*Gene amplification*

For studying EGFR gene copy numbers, DNA isolated from an aliquot of the total tissue homogenate of 25 ovarian carcinomas was digested with either Eco RI or Hind III, size fractioned on a 1% agarose gel and transferred to a nylon membrane Hybond N+ (Amersham, Buckinghamshire, UK) (Davis *et al.*, 1986). The EGFR probe was labelled by random primer extension (Feinberg & Vogelstein, 1983) using $^{32}$p-dATP. The filters were hybridised overnight at 65°C. Filters were washed at high stringency (0.3 × SSC at 65°C) and autoradiographed using Kodak XAR-5 film for 1 or 5 days at − 70°C, as described before (Berns *et al.*, 1992). Autoradiographs were scanned with a Bio-Rad Video densitometer 620. The IGF-1-receptor probe (pIGF-9-R.8, ATCC 59295) was used as a control (two gene copies) for densitometry and for the amount of DNA loaded on the gel.

*Statistics*

Associations between groups to be compared were assessed by the Spearman rank correlation test. Differences between groups was tested non-parametrically by means of the Wilcoxon two-sample test (Mann-Whitney U-test).

### Results

In non-tumorous ovarian tissues (16 patients, 21 ovaries), spindle shaped stromal cells as well as endothelial cells of vessel walls within cortical and medullary areas often showed immunoreactivity with MoAb-2E9 (in 15 out of 21 tissues, 71%) and MoAb-EGFR1 (in 18 out of 21 tissues, 85%). Moreover, moderate staining of surface epithelial cells was present in four ovaries (from three patients). Corpora albicantia and intercellular collagen did not stain with either antibody. A similar pattern was noticed in the stromal compartment of most tumour specimens (Figure 1a–c). With ligand binding assay (LBA) on membrane preparations, EGFR was measureable by Scatchard analysis in 5 out of 11 (45%) of the non-tumorous ovarian tissues examined (range: 0–50 fmol mg membrane protein).

Table I summarises the immunohistochemical (IH) data obtained with both MoAbs for 128 patients with an ovarian tumour (epithelial and non-epithelial). For all tumours analysed, a significant correlation was observed between the percentage of stained tumour cells employing MoAb-2E9 and MoAb-EGFR1 (Spearman correlation: Rs = 0.46, $n = 125$, $2P < 0.0001$). Within the tumour specimens heterogeneous levels of expression were noticed and staining was mostly cytoplasmic (Figure 1d). If a tumour was considered positive when one epithelial cell stained with one of the MoAbs, no significant difference was found in the incidence of positivity between the adenocarcinomas (primary tumours or metastases) and the other 21 benign epithelial tumours (77% *vs* 70% positive for MoAb-2E9, and 76% *vs* 77% for MoAb-EGFR1, respectively) (Table I). However, apart from one MTI tissue with MoAb-EGFR1, the maximal intensity score (*i* max; Figure 1d, e) with MoAb-2E9 and/or MoAb-EGFR1 was only observed for a varying number of cells (5–80%) in ± 12.5% of the adenocarcinoma tissues (primary tumours + metastases) examined (Table I). In 6 of these 13 tumour samples with a maximal staining intensity examined, as well as in 19 other adenocarcinomas, no amplification or rearrangement of the EGFR-gene was found by Southern blot analysis. The incidence of positivity with both MoAbs was not clearly different among the histological subtypes of the adenocarcinomas (Table II).

In the total group of adenocarcinomas, both MoAbs gave similar incidences of positivity, i.e. 75% positive for primary tumours with both MoAbs, and 86% with MoAb-2E9 and 85% with MoAb-EGFR1 for metastatic tumour samples respectively (Table I). Parallel to an increased percentage of incidence of positivity, the median level of the percentage of stained cells was higher in the metastatic lesions as compared with the primary tumours (for MoAb-2E9: 76% *vs* 36%, $2P < 0.05$; for MoAb-EGFR1: 67% *vs* 45%, $2P = 0.08$) (Table I). A similar trend was found for specimens of the primary tumours and metastatic lesions of six patients from whom both biopsies were obtained (Table III).

Regarding the non-epithelial tumour specimens, 3 (all thecomas) out of 7 did not show clear expression of EGFR with either one of the MoAbs in IH (Table I). In contrast, EGFR was detectable in both thecomas assayed with LBA (24 and 39 fmol mg membrane protein), as well as in high amounts in 1 MTI and 2 granulosa cell tumours analysed (range: 55–58 fmol mg membrane protein). For adenocarcinomas, there was a weak but statistically significant correlation between the percentage of tumour cells stained with MoAb-2E9 and with MoAb-EGFR1 (Rs = 0.37, $n = 98$, $2P < 0.001$). With LBA and with the concentration range of tracer used, EGFR was detectable by Scatchard analysis as a single class of high-affinity binding sites (Kd: 0.9 ± 0.3 nM,

**Figure 1** Non-tumorous ovarian tissue. (**a**) detail with surface epithelial cells and (**b**) spindle-shaped stromal cells and endothelial cells of vessel wall, both showing immunoreactivity for EGFR with MoAb 2E9; tumorous ovarian tissue (**c**) weak to moderate immunoreactivity with MoAb EGFR1 in some stromal cells and endothelial cells; serous carcinoma (**d**) heterogenous immunoreactivity for EGFR with MoAb 2E9 with cytoplasmic staining. Note the local grade 3 staining; detail of MTI (**e**) with grade 3 staining in epithelial cells, using MoAb EGFR1. (Indirect immunoperoxidase technique, see arrows, enlargements: **a**, 400 × ; **b**, 200 × ; **c**, 250 × ; **d**, 400 × ; **e**, 500 × .)

**Table I** Immunohistochemistry of EGFR in ovarian tumours

| Tumour type | No. of patients | MoAb-2E9 No. of positives/total (%) | MoAb-2E9 Median % positive cells | MoAb-2E9 i-max[a] | MoAb-EGFR1 No. of positives/total (%) | MoAb-EGFR1 Median % positive cells | MoAb-EGFR1 i-max |
|---|---|---|---|---|---|---|---|
| Epithelial: | | | | | | | |
| Brenner | 3 | 2/3 (67) | 95 | 0 | 3/3 (100) | 96 | 0 |
| Adenoma | 9 | 6/8 (75) | 70 | 0 | 6/9 (67) | 40 | 0 |
| Borderline | 9 | 6/9 (67) | 75 | 0 | 7/9 (78) | 60 | 0 |
| Carcinoma: | | | | | | | |
| Primary | 100 | 63/84 (75) | 36 | 10 | 62/83 (75) | 45 | 9 |
| Metastasis | | 18/21 (86) | 76[b] | 3 | 17/20 (85) | 67[c] | 4 |
| Others: | | | | | | | |
| MTI | 1 | 1/1 (100) | 100 | 0 | 1/1 (100) | 100 | 1 |
| Thecoma | 3 | 0/3 (0) | 0 | 0 | 0/3 (0) | 0 | 0 |
| Granulosa | 3 | 1/3 (33) | 100 | 0 | 3/3 (100) | 100 | 0 |

[a] i-max: number of patients with maximal staining intensity (grade 3). Mann-Whitney U-test: $2P < 0.05^{b}$ and $2P = 0.08^{c}$.

**Table II** EGFR in subtypes of primary and metastatic tumours

| Tumour type (No. of tumours) | MoAb-2E9 No. of positives/total (%) | MoAb-2E9 Median % positive cells | MoAb-2E9 i-max | MoAb-EGFR1 No. of positives/total (%) | MoAb-EGFR1 Median % positive cells | MoAb-EGFR1 i-max |
|---|---|---|---|---|---|---|
| Serous | | | | | | |
| Primary (37) | 28/37 (76) | 45 | 5 | 30/37 (81) | 40 | 4 |
| Metastasis (9) | 7/9 (78) | 70 | 3 | 8/8 (100) | 75 | 3 |
| Mucinous | | | | | | |
| Primary (20) | 15/20 (75) | 55 | 2 | 11/15 (73) | 10 | 1 |
| Metastasis (4) | 3/3 (100) | 70 | 0 | 3/4 (75) | 35 | 1 |
| Endometroid | | | | | | |
| Primary (6) | 5/6 (83) | 55 | 0 | 6/6 (100) | 52 | 1 |
| Metastasis (1) | 1/1 (100) | 60 | 0 | 1/1 (100) | 50 | 0 |
| Clear cell | | | | | | |
| Primary (8) | 6/8 (75) | 28 | 0 | 6/8 (75) | 15 | 1 |
| Metastasis (2) | 1/2 (50) | 100 | 0 | 1/2 (50) | 100 | 0 |
| Mixed | | | | | | |
| Primary (8) | 6/8 (75) | 20 | 2 | 6/8 (75) | 18 | 0 |
| Metastasis (3) | 3/3 (100) | 60 | 0 | 2/2 (100) | 52 | 0 |
| Poorly differentiated: | | | | | | |
| Primary (5) | 3/5 (60) | 15 | 1 | 3/5 (60) | 67 | 2 |
| Metastastis (3) | 3/3 (100) | 100 | 0 | 2/3 (67) | 85 | 0 |

**Table III** EGFR in primary and metastatic tumours of the same patient

| Patient number and tumour type | MoAb-2E9 Score | MoAb-2E9 Percentage stained cells | MoAb-2E9 Intensity of staining | MoAb-EGFR1 Score | MoAb-EGFR1 Percentage stained cells | MoAb-EGFR1 Intensity of staining |
|---|---|---|---|---|---|---|
| 1: primary | – | | | – | | |
| metastasis | NT[1] | | | – | | |
| 2: primary | + | 60 | 2 | + | 50 | 1 |
| metastasis | + | 70 | 2 | + | 60 | 3 |
| 3: primary | – | 10 | 1 | – | | |
| metastasis | + | 20 | 1 | + | 50 | 1 |
| 4: primary | + | 20 | 1 | – | | |
| metastasis | + | 90 | 2 | + | 20 | 1 |
| 5: primary | + | 100 | 3 | + | 90 | 2 |
| metastasis | + | 60 | 2 | NT[1] | | |
| 6: primary | – | | | – | | |
| metastasis | + | 100 | 2 | + | 15 | 2 |

[1] Not tested.

mean ± s.d.) in 66% (48/73; median 17, range: 0–158 fmol mg membrane protein) of the tumours analysed. A statistically significant correlation was noted between the levels of EGFR assessed with LBA and the percentage of stained tumour cells determined immunohistochemically with MoAb-EGFR1 (Rs = 0.59, $n = 71$, $2P < 0.0001$). However, the relationship between EGFR measured by LBA with that of MoAb-2E9 was far weaker (Rs = 0.21, $n = 73$, $2P < 0.1$).

We have subsequently chosen arbitrary cut-off points to distinguish between EGFR-positive and -negative, in such a way that in each case approximately two-thirds of the tumours were positive. For these 73 adenocarcinoma biopsies and using $> 0$ fmol mg membrane protein as cut-off point for EGFR-positivity assessed by LBA, and 10% stained cells as cut-off point for IH with both MoAbs, similar percentages of positivity were observed, i.e. 66% for LBA, 70% for MoAb-2E9, and 66% for MoAb-EGFR1. The lowest accordance was found for data obtained with LBA and MoAb-2E9 (45% discordance). Data obtained with both MoAbs also showed a relatively low accordance (34% discordance), whereas the

**Figure 2** Comparison ligand-binding assay with IH for EGFR with MoAb 2E9 (**a**) and MoAb EGFR1 (**b**).

accordance between data obtained with LBA and MoAb-EGFR1 was highest (only 28% discordance). There was no significant difference in the percentage of stained cells with MoAb-2E9 in tumours positive or negative for EGFR as assessed by LBA (Figure 2, left). On the other hand, the percentage of positive tumour cells determined by IH with MoAb-EGFR1 was significantly higher in EGFR-positive tumours assayed by LBA as compared with those which lacked specific $^{125}$I-EGF binding ($2P<0.0001$; Figure 2, right).

### Discussion

This study was undertaken to investigate the correlation between EGFR and ovarian tumour type in a series of 128 patients, using two MoAbs, one reactive to the low-affinity ligand binding class of EGFR (MoAb-3E9), and one reactive to both high- and low-affinity EGFR (MoAb-EGFR1), and to compare these IH data with LBA and EGFR gene amplification. We have shown that comparing the IH data of MoAb-2E9 with those of MoAb-EGFR1, when applied on all epithelial and non-epithelial tumour samples, there was a significant correlation between the percentage of stained tumour cells ($2P<0.0001$). With either MoAb, 77% of the adenocarcinoma samples (primary and metastatic tumours) collected from 100 patients, stained positive for EGFR. This incidence of positivity is in agreement with the results reported by Battaglia *et al.* (1989; 75% EGFR-positive in 24 cases) and by Berchuck *et al.* (1991; 77% EGFR-positive in 87 cases), where were obtained using LBA and IH respectively, but higher than those reported by Bauknecht *et al.* (1990); 50% EGFR-positive in 222 cases), who used both techniques, and Owens *et al.* (1991; 39.7% EGFR-positive in 199 cases), who used LBA. The IH results in the present study showed within-specimen heterogeneity of the levels of expresson (from 5 to 100% of the cells). In 12.5% of the adenocarcinomas overexpression (intensity score $i$ max = 3) was observed in some of the tumour cells. Overexpression of EGFR protein might possibly be caused by amplification of the EGFR gene. However, no EGFR gene amplification was found in any of the 25 ovarian tumours studied, not even in the six tumours showing high expression of EGFR protein by IH. The absence of amplification of the EGFR gene in ovarian tumour biopsies was also reported by Bauknecht *et al.* (1990), Gullick *et al.* (1986), and Zhang *et al.* (1989). Therefore, amplification of the EGFR gene is not likely to be involved in ovarian carcinogenesis.

We found an increased incidence of positivity and median percentage of stained cells in metastatic lesions, as compared with primary tumours. Similar results were reported by Battaglia *et al.* (1989), but these findings were not confirmed by others (Bauknecht *et al.*, 1990; Berchuck *et al.*, 1991). No significant difference was observed in the incidence of EGFR-positivity among the histological subtypes of adenocarcinomas (Table II), as was also suggested before by Witmaack *et al.* (1988). In general, the staining was cytoplasmic for both MoAbs, as has also been reported by others (Damjanov *et al.*, 1986; Defize *et al.*, 1986; Rodriquez *et al.*, 1991; Berchuck *et al.*, 1991). Localisation of receptors in the cytoplasmic compartment may reflect internalisation of receptor, a rapid process that occurs after ligand binding. On the other hand, it may represent a mechanism by which postmitotic cells maintain the capacity to bind EGF, and escape the acute mitogenic signal in the presence of circulating EGF (or TGF-$\alpha$) (Damjanov *et al.*, 1986).

Comparing our IH data with those of LBA on adenocarcinoma samples, some surprising results were obtained. EGFR was detectable by Scatchard analysis in 66% of the tumours analysed, and a statistically significant correlation was found between the level determined with LBA and the percentage of cells stained with MoAb-EGFR1 ($2P<0.0001$). In contrast, the relationship between biochemically assessed EGFR and the percentage of stained tumour cells with MoAb-2E9 was very weak ($2P<0.1$). When comparing non-tumours ovarian tissues with adenocarcinomas using LBA, the frequency of EGFR-positivity was higher in the adenocarcinomas (66% *vs* 45%). This difference between the incidence of positivity was not found when using IH with either MoAb. Moreover, a significant number of discordances was observed when comparing EGFR status as assessed with LBA and with IH, particularly with MoAb-2E9. This might be explained by heterogeneity of receptor distribution in the tumour tissue. However, the low correlation between LBA and IH for MoAb-2E9 is more likely to be caused by the fact that with LBA in the concentration range of ligand used, probably only the high-affinity class of EGFR was determined, whereas MoAb-2E9 detects only the low-affinity class of EGFR (Defize *et al.*, 1989).

The 28% discordances observed between LBA and MoAb-EGFR1 may not just be caused by heterogeneity in tissue distribution of EGFR but also by the presence of EGFR-

positive stromal-derived membranes, causing EGFR-positivity in LBA and EGFR-negativity with IH using MoAb-EGFR1 when scoring only epithelial cells. This latter possibility is not likely, as the stromal compartment of tumours which scored positive in LBA and negative with IH was negative for EGFR with MoAb-EGFR1. Some of the discordances may have been caused by the presence of receptors with an intact antigenic site, scoring positive with IH but negative with LBA, as they are unable to bind ligand. However, to our knowledge no such receptors unable to bind ligand have been described in the literature for any tumour tissue. It is important to keep in mind that with LBA only EGFR localised in the crude membrane preparation was determined and that with IH cytoplasmic staining was most frequently observed in all specimens. Thus the two techniques detect receptors at entirely different subcellular localisations, and it may therefore be unrealistic to expect full concordances between LBA and IH.

In summary, although highly significant correlations were found between the levels of EGFR measured by LBA as compared with the percentage of positive tumour cells stained immunohistochemically with MoAb-EGFR1, at least in part different EGFR entities are probably determined by both techniques. Clinical studies with the lengths of relapse-free and overall survival as parameters are necessary to establish the possible prognostic impact of EGFR determined with either methodology or whether a combination of both will give a better discrimination of high- and low-risk patients. Such a study is currently in progress.

We gratefully express our thanks to the pathologists from all collaborative Pathology departments of the following Hospitals: Zuiderziekenhuis, Clara Ziekenhuis, Academisch Ziekenhuis Dijkzigt, PATHAN, all located in Rotterdam, and Maria Ziekenhuis in Tilburg and the Pathology Department in Bergen op Zoom, who generously provided us with ovarian (tumour) tissues.

The authors wish to thank Elly Fieret, Henk Portengen, Erica Noordegraaf, Iris van Staveren and Marly Stuurman-Smeets for their expert technical assistance, and Anja van Weeszenberg for editing the text.

Supported by the Dutch Cancer Society (grant DDHK 90-05).

### References

BATTAGLIA, F., SCAMBIA, G., BENEDETTI PANICI, P., BAIOCCHI, G., PERRONE, L., IACOBELLI, S. & MANCUSO, S. (1989). Epidermal growth factor receptor expression in gynaecological malignancies. *Gynecol. Obstet. Invest.*, **27**, 42-44.

BAUKNECHT, Th., RUNGE, M., SCHWALL, M. & PFLEIDERER, A. (1988). Occurrence of epidermal growth factor receptors in human adnexal tumours and their prognostic value in advanced ovarian carcinomas. *Gynecol. Oncol.*, **29**, 147-157.

BAUKNECHT, Th., JANZ, I., KOHLER, M. & PFLEIDERER, A. (1989). Human ovarian carcinomas: correlation of malignancy and survival with the expression of epidermal growth factor receptors (EGF-R) and EGF-like factors (EGF-F). *Med. Oncol. Tumor Pharmacother.*, **6**(2), 121-127.

BAUKNECHT, Th., BIRMELIN, G. & KOMMOSS, F. (1990). Clinical significance of oncogenes and growth factors in ovarian carcinomas. *J. Steroid Biochem. Molec. Biol.*, **37**(6), 855-862.

BENRAAD, Th.J. & FOEKENS, J.A. (1990). Hydroxyapatite assay to measure epidermal growth factor receptor in human primary breast tumours. *Ann. Clin. Biochem.*, **27**, 272-273.

BERCHUCK, A., RODRIQUEZ, G., KAMEL, A., DODGE, R.K., SOPER, I.T., CLARKE-PEARSON, D.L. & BAST, R.C. (1991). Epidermal growth factor receptor expression in normal ovarian epithelium and ovarian cancer I. Correlation of receptor expression with prognostic factors in patients with ovarian cancer. *Am. J. Obstet. Gynecol.*, **164**, 669-674.

BERNS, E.M.J.J., KLIJN, J.G.M., VAN PUTTEN, W.L.J., VAN STAVEREN, I.L., PORTENGEN, H. & FOEKENS, J.A. (1990). c-Myc amplification is a better prognostic factor than HER2/neu amplification in primary breast cancer. *Cancer Res.*, **52**, 218-224.

DAMJANOV, I., MILDNER, B. & KNOWLES, B. (1986). Immunohistochemical localization of the epidermal growth factor receptor in normal human tissues. *Lab. Invest.*, **55**(5), 588-592.

DAVIS, L.G., DIBNER, M.D. & BATTEY, J.F. (1986). *Basic Methods in Molecular Biology*. Elsevier: New York.

DEFIZE, L.H.K., MOOLENAAR, W.H., VAN DER SAAG, P.T. & DE LAAT, S.W. (1986). Dissociation of cellular responses to epidermal growth factor using anti-receptor monoclonal antibodies. *EMBO J.*, **5**, 1187-1192.

DEFIZE, L.H.K., BOONSTRA, J., MEISENHELDER, J., KRUYER, W., TERTOOLEN, L.G.J., TILLY, B.C., HUNTER, T., VAN BERGEN EN HENEGOUWEN, P.M.P., MOOLENAAR, W.H. & DE LAAT, S.W. (1989). Signal transduction by epidermal growth factor occurs through the subclass of high affinity receptors. *J. Cell Biol.*, **109**, 2495-2507.

E.O.R.T.C. BREAST CANCER COOPERATIVE GROUP (1980). Revision of the standards for the assessment of hormone receptors in human breast cancer. *Eur. J. Cancer*, **16**, 1513-1515.

FEINBERG, A.P. & VOGELSTEIN, B. (1983). A technique for labelling DNA restriction endonuclease fragments to high specific activity. *Anal. Biochem.*, **132**, 6-13.

GULLICK, W.J., MARSDEN, J.J., WHITE, N., WARD, D., BOBROW, L. & WATERFIELD, M.D. (1986). Expression of EGF-receptors on human cervical, ovarian and vulval carcinomas. *Cancer Res.*, **46**, 285-292.

HENZEN-LOGMANS, S.C., VAN DEN BURG, M.E.L., FOEKENS, J.A., BERNS, P.M.J.J., BRUSSÉE, R., FIERET, J.H., KLIJN, J.G.M., CHADHA, S. & RODENBURG, C.J. (1992). Occurrence of epidermal growth factor receptors (EGF-R0 in various benign and malignant ovarian tumors and normal ovarian tissues: An immunohistochemical study. *J. Cancer Res. Clin. Oncol.*, **118**, 1-5.

KIENHUIS, C.B.M., HEUVEL, J.J.T.M., ROSS, H.A., SWINKELS, L.M.J.W., FOEKENS, J.A. & BENRAAD, Th.J. (1991). Six methods for direct radioiodination of mouse epidermal growth factor compared: Effect of noequivalence in binding behaviour between labeled and unlabeled ligand. *Clin. Chem.*, **37**, 1749-1755.

KLIJN, J.G.M., BERNS, P.M.J.J., SCHMITZ, P.I.M. & FOEKENS, J.A. (1992). The clinical significance of epidermal growth factor receptor (EGF-R) in human breast cancer: A review on 5232 patients. *Endocrinol. Rev.*, **13**, (in press).

KOENDERS, P.G., BEEX, L.V.A.M., GEURTS-MOESPOT, A., HEUVEL, J.J.T.M., KIENHUIS, C.B.M. & BENRAAD, Th.J. (1991). Epidermal growth factor receptor-negative tumors are predominantly confined to the subgroup of estradiol receptor-positive human primary breast cancers. *Cancer Res.*, **51**, 4544-4548.

NICHOLSON, S., SAINSBURY, J.R.C., HALCROW, P., CHAMBERS, P., FARNDON, J.R., HARRIS, A.L. (1989). Expression of epidermal growth factor receptors associated with lack of response to endocrine therapy in recurrent breast cancer. *Lancet*, **i** 182-185.

OWEN, O.J., STEWART, C., BROWN, I. & LEAKE, R.E. (1991). Epidermal growth factor receptors (EGFR) in human ovarian cancer. *Br. J. Cancer*, 64, 907-910.

RODRIQUEZ, G., BERCHUCK, A., WHITACKER, R.S., SCHLOSSMAN, D., CLARKE-PEARSON, D. & BAST, R.C. (1991). Epidermal growth factor receptor expression in normal ovarian epithelium and ovarian cancer II. Relationship between receptor expression and response to epidermal growth factor. *Am. J. Obstet. Gynecol.*, **164**, 745-755.

SAINSBURY, J.R.C., FARNDON, J.R., NEEDHAM, G.K., MALCOLM, A.J. & HARRIS, A.L. (1987). Epidermal growth receptor status as predictor of early recurrence of and death from breast cancer. *Lancet*, **ii**, 1398-1402.

SCHERES, H.M.E., DE GOEIJ, A.F.P.M., ROUSCH, H.J.M., HONDIUS, G.G., WILLEBRAND, D.D., GIJZEN, H.H. & BOSMAN, F.T. (1988). Quantification of oestrogen receptors in breast cancer: radiochemical assay on cytosols and cryostat sections compared with semiquantitative immunocytochemical analysis. *J. Clin. Pathol.*, **41**, 623-632.

SEROV, S.F., SCULLY, R.E. & SOBIN, L.H. (1973). *International Histological Classification of Tumours, NOG. Histological Typing of Ovarian Tumours*. World Health Organisation: Geneva.

TAYLOR-PAPADIMITRIOU, J., SHEARER, M. & STOPKER, M.G.P. (1977). Growth requirements of human mammary epithelial cells in culture. *Int. J. Cancer*, **20**, 903–908.

WITMAACK, F., SCHWORER, D., WINTZER, O., BAUKNECHT, Th. & PFLEIDERER, A. (1988). The immunohistochemical expression of epidermal growth factor receptors in various gynaecological tumors. *J. Cancer Res. Clin. Oncol.*, **114**(suppl), 114–115.

ZHANG, X., SILVA, E., GERSHENSON, D. & HUNG, M. (1989). Amplification and rearrangement of c-erbB protooncogens in carcinomas of human female genital tract. *Oncogene*, **4**, 985–989.